# Thematic areas and complexity of integrated community case management (iCCM) design, implementation, and evaluation: protocol for a scoping review

**DOI:** 10.1186/s13643-020-01454-y

**Published:** 2020-09-03

**Authors:** Aliya Karim, Daniel Cobos Muñoz, Daniel Mäusezahl, Don de Savigny

**Affiliations:** 1 The Swiss Tropical and Public Health Institute (Swiss TPH), Socinstrasse 57, 4051 Basel, Switzerland; 2 The University of Basel, Basel, Switzerland

**Keywords:** iCCM, Integrated community case management, Malaria, Pneumonia, Diarrhea, Scoping review, Health systems research

## Abstract

**Background:**

Integrated community case management (iCCM) is a community-based child health strategy designed to reduce deaths due to pneumonia, malaria, and diarrhea in low-income countries. Due to the integrated nature of the intervention and the diversity of its stakeholders and activities, iCCM is complex and comprises many systems elements. However, the extent to which studies examine these different elements is unknown. The purpose of this scoping review is to summarize the key areas of emphasis of the iCCM literature and assess the extent to which this takes into account systems complexity.

**Methods:**

This study will be guided by Arksey and O’Malley’s scoping review methodology. We will systematically screen MEDLINE, Web of Science, and the specialized platform Community Case Management (CCM) Central Library for published literature in English related to the design, implementation, and evaluation of iCCM. Two investigators will independently screen the full list of titles and abstracts for eligibility, followed by a full-text review of selected titles divided between investigators. Emergent themes will be categorized according to a thematic tool iteratively developed to guide the charting and analysis process. To compare the extent to which the literature assesses systems factors, we will compare our results with the iCCM Interagency Framework. We will use the Intervention Complexity Tool for Systematic Reviews (iCAT_SR) to assess how literature measures complexity. Results will be presented in narrative fashion, supplemented by interactive graphical interfaces.

**Discussion:**

The results of this scoping review will identify the priorities and deficiencies of the analysis and evaluation of iCCM programs and may illustrate the need for systems approaches. Bottom-up emergent iCCM themes can help researchers, policymakers, and implementers target and better emphasize true priorities of iCCM. Understanding how complexity is considered and examined in iCCM may result in greater attention to this critical dimension of iCCM program assessment, resulting in the design and development of more robust and sustainable iCCM programs.

## Background

Global, over five million children died in 2018 before the age of five. More than half of these deaths were considered preventable with access to simple and inexpensive treatment; however, accessibility to healthcare services remains one of the most pervasive challenges to reducing child mortality [1, 2]. Integrated community case management (iCCM) is a strategy that aims to address this through community-based diagnosis and care of common childhood illnesses in hard-to-reach areas of low- and middle-income countries (LMICs). The intervention is implemented by local community health workers (CHWs) within assigned catchment areas, who are trained and equipped to diagnose and treat a range of conditions responsible for high child mortality rates, particularly pneumonia, malaria, and diarrhea [3–5]. iCCM was globally endorsed in 2004 in a joint statement produced by the United Nations Children’s Fund (UNICEF) and the World Health Organization (WHO). Since then, it has been adopted by many countries, including most of sub-Saharan Africa, as a common child health policy [6–9].

iCCM is generally considered a relatively complex intervention, comprising many interactions among multiple processes and actors across a range of administrative levels and systems building blocks. These elements are wide in scope and can include anything from the procurement and distribution of supplies, country policy environments, supervision strategies, and design inputs, to caregiver and CHW interactions, and geographic contexts, among others [10–12]. A number of these elements have been considered in the development of global benchmarks for the rollout and monitoring of iCCM [13]. However, much of the literature analyzing iCCM as an applied strategy is relatively recent, and the extent to which this addresses or measures these different dimensions of design and implementation is not completely known. Moreover, while these global benchmarks have been tested for feasibility in collection, these have not to our knowledge been tested for comprehensiveness to evaluate the design, implementation, and monitoring of iCCM [14].

Another limitation to these benchmarks is how iCCM was initially developed. Results from a compendium of studies evaluating the evolution of iCCM policy enterprises by Bennet et al. revealed that the prioritization and development of iCCM as a widely accepted child health strategy were largely guided by a group of individuals who acted cyclically as researchers, knowledge-brokers, global policy influencers, and program managers, and within a relatively short period of time [15–18]. This new evidence demonstrated how priorities for the structure, execution, and evaluation of iCCM can be largely influenced and echoed among the same community of stakeholders, limiting the potential of inputs from downstream actors and results from operational research to guide critical areas of emphasis. This consequently exposes the potential for disconnect not only between what is prioritized among such epistemic communities and what is emphasized in operational research, but also what is considered to be of more or differing relevance according to emergent data and voices from the field. Finally, while iCCM is an inherently complex intervention reliant upon a number of interactions and codependent systems elements, the extent to which the current literature assesses this complexity is not known.

We argue that a comprehensive approach to iCCM assessment incorporates the evaluation of systems complexity and of how systems elements interact with each other within a dynamic environment. Such approaches can broaden global targets for the iCCM scale, heighten policymakers’ and implementers’ sensitivity to the interconnected nature of the intervention to enable more robust implementation, and engender sustainability. This scoping review aims to (i) map the current iCCM literature landscape by allowing the breadth of selected studies to populate an emergent list of iCCM components and respective indicators; (ii) compare how these correspond to global priorities for iCCM, identifying gaps in existing benchmarks; and (iii) measure how complexity is assessed in these studies. This is the first scoping review to our knowledge that addresses iCCM with a systems lens.

## Methods

We will conduct a scoping review to map the extent, scope, and complexity of iCCM literature. This study protocol is not registered with PROSPERO as scoping reviews are currently not eligible for registration. The methodology for this scoping review will be based on five key phases of the standardized scoping review approach developed by Arksey and O’Malley and elaborated by Levac et al. [19, 20]. These stages are (i) determining the research question, (ii) identifying relevant literature, (iii) selecting studies, (iv) charting the data, and (v) collation, summary, and reporting results. Common practice for systematic reviews employs the PRISMA-P or PRISMA-ScR checklist; the latter is used to guide this scoping review (Additional File 1). Steps 4 and 5 will take advantage of the standard framework approach in the systematic coding, categorization, and synthesis of emergent themes across the literature. As an additional step, we will conduct a complexity assessment using the Intervention Complexity Tool for Systematic Reviews (iCAT_SR) tool to measure how studies take into account systems dynamics and complexity in their design and/or evaluation.

### Determining the research question and objectives

The primary purpose of this scoping review is to consolidate and map existing peer-reviewed, published literature on iCCM to determine the thematic areas describing its design, implementation, monitoring, and evaluation as a complex child health intervention, and identify research priorities and outlying gaps to better inform programmers, researchers, and policymakers. We are guided by three key research questions:

1. What are the key thematic systems areas of emphasis according to the current body of evidence available on iCCM?
2. How well do we do these align with existing global standards for the design, implementation, and evaluation of iCCM, and what gaps exist between these?
3. To what extent does the current literature assess intervention complexity and health systems dynamics?

Our objectives are to (i) develop a taxonomic categorization of iCCM themes and categories and map these accordingly, (ii) assess the extent to which these core themes of intervention research are represented, and (iii) measure how systems integration and complexity are analyzed or assessed in any of the studies.

### Identifying relevant studies and search strategy

An in-house research librarian was consulted in the design of our search strategy to obtain conceptually relevant papers. We will systematically search peer-reviewed, published studies to generate a set of literature meeting defined eligibility criteria. Gray literature will be excluded from this review, as we are interested in the research foci of peer-reviewed, publically accessible literature. Our search will be conducted using the following databases and sources:

- Healthcare, medical, and scientific journal repositories MEDLINE (PubMed) and Web of Science
- Specialized knowledge platforms including the Community Case Management Central Library
- Manual searching of known or targeted journals and supplements (i.e., *Ethiopian Medical Journal*)
- Publication lists from relevant articles identified

Databases such as EMBASE are not included in our search as it is primarily a pharmacological database, which falls outside the main scope of iCCM. We will search these databases using keywords related to iCCM. Because of this requirement, iCCM or community case management (CCM) as a concept or strategy must be explicitly named within the publication. CCM is a precursor to the term iCCM and may be used interchangeably with iCCM. The search terms to be used are the following keywords and Boolean operators:

- “integrated community case management” OR
- iCCM OR
- “community case management”

Zotero and Endnote bibliographic software will be used to import, organize, and screen articles.

### Inclusion and exclusion criteria

#### Inclusion criteria

Any peer-reviewed publication that examines iCCM in any form as a strategy or concept, or focuses on a critical component of that strategy with the aim to inform iCCM, is considered for inclusion in the review. iCCM for the purposes of this review is defined as the standard package of integrated services provided by CHWs, primarily targeting pneumonia, malaria, and diarrhea in children under 5 years directly in hard-to-reach areas of LMICs. Critical components of iCCM can include any part of the iCCM intervention without assessing the intervention as a whole, such as its commodities and drug delivery mechanisms, training of human resources, data collection strategies, and service delivery algorithms, among many others. This can include both empirical studies and conceptual papers which may not analyze an iCCM program, but rather its surrounding supportive environment, policies relating to it, or existing primary healthcare structures that may influence how iCCM is designed, implemented, or sustained. However, these must make reference to iCCM. For example, a study which assesses how maternal and child health (MCH) policies have been generally successful in a particular country would fall outside the inclusion criteria. This is because while MCH is relevant to iCCM as an approach to child health, this must be explicitly related to its influence on or the influence of iCCM; otherwise, this would not be considered eligible. Therefore, included literature must:

- Be a structured, peer-reviewed publication in English emanating from a reputable source indexed by one of the databases included in the search strategy
- Examine iCCM or a component of the strategy, where attribution is explicitly linked to iCCM
- Have been published after 2002

Any quantitative or qualitative studies are eligible, except for those which fall outside exclusion criteria (below). Studies of iCCM as a pilot program are considered inclusion criteria. Each peer-reviewed publication is treated as a separate study, irrespective of whether or not these examine the same iCCM program or overlapping results. Because iCCM is only limited to implementation in LMICs, any country implementing iCCM is eligible for inclusion. We will only search articles published after 2002 as references to iCCM as a defined program occurred after this year [4–6].

#### Exclusion criteria

Articles that are not at least partially focused on assessing iCCM as a package of services according to its classical definition of an integrated child health intervention targeting multiple childhood illnesses implemented within communities of hard-to-reach areas of low- and middle-income countries (LMICs) will be excluded. Articles that do not discuss, examine, assess, or analyze iCCM as a policy, intervention, or component of an intervention will also be excluded, as will articles simply mentioning or discussing CCM or iCCM in a broader context, unless assessments are conducted in preparation for the implementation or scale-up of iCCM. Analyses of non-integrated vertical programs (i.e., malaria-specific, severe acute malnutrition (SAM)-only) will be omitted. Studies focusing on CHWs which are not explicitly involved in an iCCM program in some capacity, or those assessing iCCM-practicing CHWs where analysis cannot be attributable to the iCCM intervention, do not meet inclusion criteria. Studies assessing general regional trends (i.e., demographic health surveys) that are not focused on iCCM program impact, even if study areas are implementing iCCM, will be ignored. Studies assessing caregiver attitudes towards CHWs will be included only if CHWs were iCCM-practicing at the time of study or are expected to be, or factors influencing caregiver attitudes or behavior are directly attributable to the iCCM intervention.

The following document types will be excluded:

- Non-peer-reviewed documents including:
  - Strategy documents, policy documents, joint statements
  - Treatment guidelines, training or implementation manuals, materials or protocols
  - Roadmaps or sustainability plans
  - Systematic reviews
  - Descriptive documents and information notes
- Articles not directly assessing, analyzing, or discussing iCCM programs according to the standard definition of iCCM

While systematic reviews are excluded, we will check for any relevant studies used and include them if they satisfy the above criteria.

### Selecting the literature

We will independently review papers for duplicates and relevance according to defined inclusion and exclusion criteria. Titles and abstracts will first be screened independently by two investigators, and after a subset of papers have been identified, both will perform a full-text review where papers are divided between investigators. Outstanding issues over the inclusion of studies will be resolved among the remaining investigators on the team alongside both investigators. Because themes are emergent according to overarching category and domain, any new themes that differ according to subgroups are categorized accordingly on an iterative basis.

### Classification of studies

The classification process will use a thematic coding analytic method to cluster areas or categories found in selected publications. Because categories are expected to iteratively emerge from the literature, and are also guided by preconceptions of expected themes, we will use the Framework Method as an inductive-deductive approach to classification [21]. While the Framework Method is conventionally applied to qualitative transcripts, here, we use its basic premise as a systematic method to group and synthesize data elements according to hierarchical relevance. Data extraction matrices have been developed to collect information on these thematic areas. These thematic areas and categories will be iteratively added, condensed, and re-defined according to the extraction process. Because of this iterative process, the data extraction tool will not be pilot tested. To ensure comprehensiveness and reproducibility of categorization, two investigators will conduct a final round of verification, where any outstanding issues related to extraction will be resolved across the team.

### Data items and charting domains

Data extraction is subdivided into six overarching domains: (i) publication profile, (ii) core study information, (iii) iCCM program architecture, (iv) iCCM systems dimensions, (v) implementation outcomes, and (vi) complexity. These domains capture the key elements of the review studies for mapping their shared characteristics, extracting thematic areas for coding, identifying key iCCM systems elements for comparison against an existing framework, and determining priority areas of research and existing gaps in the literature. Table 1 lists these six domains and their corresponding categories. The publication profile collects general publication information, including publication year, author(s), and journal. Core study information defines the purpose, design, methods, and background of the study and includes information on the variables used for measurement, level of assessment or study participant(s), funders, and collaborating partners managing the study. The iCCM program architecture domain collects information on the design and structure of the evaluated iCCM program, which can help elucidate the typography of iCCM programs and the variations they can take from a core design.

**Table 1:** Data extraction instrument

Domain no.	Domain	Category
**I**	**Publication profile**	Article title; author(s); publication year; journal; DOI
**II**	**Core study information**	Year(s) of study; purpose of study; main findings; study type or design; data type: quantitative, qualitative, or mixed; study level(s) (caregiver, CHW, administrative division); disease concentration; variables used and defined; measured outcomes; funder(s) of study; collaborating institutions (authors)
**III**	**iCCM program architecture**	Country/ies of iCCM implementation; program name; duration of iCCM implementation; entities implementing iCCM; funder(s) of iCCM; CHW density and distribution; inputs, tools, and logistics; MoH roles; management and partner roles
**IV**	**iCCM systems dimensions**	*Emergent categories to be determined through charting process*
**V**	**Implementation outcomes**	Effectiveness; coverage; sustainability; feasibility; acceptability; adoption; appropriateness; cost; fidelity; penetration; efficiency; equity; quality; timeliness
**VI**	**Complexity assessment**	*Numerical scores based on four dimensions of complexity*

The fourth domain, iCCM systems dimensions, is purposefully left open to allow selected studies to guide the charting process inductively. Possible elements that would fall under thematic categories within this domain could include stock outs and commodities, CHW motivation, care seeking, forms and data transmission, mHealth, iCCM policy, costing, supervision, and training, among many others. We envisage that this will entail elements involving critical processes of iCCM, the way that actors relate to each other and perform activities, and how existing contexts may influence iCCM rollout.

The fifth domain, implementation outcomes, identifies which implementation outcomes are examined or measured in selected studies. These constructs are generally used to evaluate different facets of program success, where outcomes used in this review are based on those identified within the conceptual framework of implementation indicators posited by Proctor et al. [22]. Table 2 provides these indicators and their definitions.

**Table 2:** Implementation and service outcomes

Implementation and service outcome	Definition
**Acceptability**	The perception among implementation stakeholders that a given treatment, service, practice, or innovation is agreeable, palatable, or satisfactory.
**Adoption**	The intention, initial decision, or action to try or employ an innovation or evidence-based practice. Adoption also may be referred to as “uptake.”
**Appropriateness**	The perceived fit, relevance, or compatibility of the innovation or evidence-based practice for a given practice setting, provider, or consumer, and/or perceived fit of the innovation to address a particular issue or problem.
**Costs**	The cost impact of an implementation effort, including itemized costs of administration overheads, commodities and supplies, and human resources.
**Coverage**	The extent to which an intervention provides services to the target population.
**Effectiveness**	How well the applied intervention or innovation successfully produces or influences the desired outcome.
**Efficiency**	How optimally resources are applied in the implementation of an intervention or innovation, or to what extent the intervention or innovation produces a desired output relative to its time and resource expenditure.
**Equity**	The absence of remedial differences in the delivery and reception of services of an intervention among its target population.
**Feasibility**	The extent to which a new treatment, or an innovation, can be successfully used or carried out within a given agency or setting.
**Fidelity**	The degree to which an intervention was implemented as it was prescribed in the original protocol or as it was intended by the program developers.
**Penetration**	The integration of a practice within a service setting and its subsystems.
**Quality**	The extent to which an intervention and its services are effective, timely, patient-centered, and safe. Quality can be considered a composite indicator of various dimensions and service indicators.
**Sustainability**	The extent to which a newly implemented treatment is maintained or institutionalized within a service setting’s ongoing, stable operations.
**Timeliness**	How rapidly an intervention or service addresses the intended target population or patient within an appropriate timeframe.

#### Complexity assessment

iCCM is an inherently complex intervention, and taking this complexity into account is considered critical for holistic assessments. We therefore adapt a tool to treat complexity within the sixth domain: to measure how the literature addresses, interprets, or includes concepts of complexity in their examination of iCCM or the components that comprise it. We employ the iCAT_SR tool, an instrument developed to measure the complexity of interventions by building on pre-defined concepts of complexity [23–25]. While the iCAT_SR tool is primarily used to facilitate the systematic assessment of the complexity of interventions themselves, as opposed to the extent to which a study accounts for this complexity, the scoring metrics of the tool can be applied within the scope of this review. We will use four of the seven dimensions defined in the tool to generate a composite numeric score of complexity of how studies examine or measure complexity. Each core dimension is given a score of “1” if the publication fulfills the criteria of that dimension; otherwise, the score is zero. The sum total aggregates these scores across the four dimensions. Table 3 lists these dimensions and their binary scoring metrics.

**Table 3:** iCAT_SR complexity scoring

Domain	Core dimension of iCAT_SR tool	Score
**Study complexity**	Assesses or measures the degree of interaction between intervention components, including the independence/interdependence of intervention components	1
	Assesses or measures the degree to which the effects of the intervention are dependent on the context or setting in which it is implemented	1
	Assesses or measures the degree to which the effects of the intervention are changed by recipient or provider factors	1
	Assesses or examines the nature of the causal pathway between the intervention and the outcome it is intended to effect	1
	*Maximum Total*	4

#### Systems dimensions assessment

To assess the extent to which the breadth of iCCM literature examines system elements prioritized by global mandates and iCCM epistemic communities, we will compare the results of the scoping review against an existing iCCM systems benchmark framework [13]. The USAID iCCM Interagency Framework is a series of thematic benchmarks and indicators developed by a technical working group of iCCM experts that lists the primary systems elements believed to be critical to the design, implementation, and monitoring of iCCM programs. We aim to compare how well these domains are represented in the existing literature, and identify gaps which may be prioritized by evidence but deficient in the Interagency Framework, or vice versa.

### Data synthesis

The coded constructs emerging from review findings will be used to guide knowledge synthesis, which will occur according to standard methodology [21, 26, 27]. Thematic elements extracted from studies will be categorized according to the standard framework approach and mapped according to corresponding and overlapping themes. Articles will be summarized using tables in Excel according to the pre-defined categories, where emerging iCCM systems dimensions will be added iteratively to the framework.

### Quality assessment

We will not exclude studies based on quality, as the aim of the review is to chart available published evidence. However, we will review the quality of methods used in studies included in this review using the requisite Joanna Briggs Institute (JBI) critical appraisal tool [28] to evaluate the methodological robustness of included studies. We will follow the PRISMA-ScR checklist to ensure each step in the scoping review process is standardized.

### Reporting results

Results will be reported in a format to suit a variety of audiences. We will provide a tabular interface listing all reviewed literature and their corresponding thematic areas and characteristics listed in the “Classification of studies” section and the information provided in Tables 1, 2, and 3. To illustrate reported findings, we will also produce a heat map of the countries of focus and their basic iCCM design characteristics, as well as a social network analysis map of the authors publishing articles included in this review. Interactive web or radial diagrams will present the various emerging systems dimensions of iCCM, and the extent to which the literature addresses these dimensions. These dimensions and their core findings will be described and key findings presented. These will help build a basis for an iCCM systems conceptual framework. Finally, complexity scores assigned to publications will be presented in graphical interface. These diagrams will be included in the review as either illustrative graphics or appendices, while interactive versions will be freely available as online dashboards. The framework of products can also assist in developing potential systematic reviews assessing outcomes within a particular thematic category.

## Discussion

To our knowledge, this is the first scoping review of iCCM to assess the breadth of available iCCM research. With this scoping review, we aim to provide a comprehensive assessment of the critical themes necessary to the successful design, implementation, and evaluation of iCCM, and guide future comprehensive evidence building for the intervention.

## Supplementary information

**Additional file 1:** PRISMA-ScR Checklist.

## Data Availability

Not applicable.

## Abbreviations

CCM: Community case management
CHW: Community health worker
iCAT_SR: Intervention Complexity Tool for Systematic Reviews
iCCM: Integrated community case management
JBI: Joanna Briggs Institute
LMIC: Low- and middle-income country
MCH: Maternal and child health
SAM: Severe acute malnutrition
Swiss TPH: Swiss Tropical and Public Health Institute
UNICEF: United Nations Children’s Fund
WHO: World Health Organization
